# The Perception of Environmental Information Disclosure on Rural Residents’ Pro-Environmental Behavior

**DOI:** 10.3390/ijerph19137851

**Published:** 2022-06-26

**Authors:** Yongliang Yang, Yuting Zhu, Xiaopeng Wang, Yi Li

**Affiliations:** 1 School of Economics and Management, Zhejiang Sci-Tech University, Hangzhou 310018, China; royyang@zju.edu.cn (Y.Y.); 2019333503089@mails.zstu.edu.cn (Y.Z.); 2 Silk and Fashion Culture Research Center of Zhejiang Province, Zhejiang Sci-Tech University, Hangzhou 310018, China; 3 Fashion Department, DongHai Academy, Collaborative Innovation Center of Port Economy, Ningbo University, Ningbo 315211, China

**Keywords:** rural environmental issues, environmental information disclosure, pro-environmental behavior, personal environmental concern

## Abstract

Rural residents’ pro-environmental behavior plays a critical role in rural environmental governance. This paper examines how the perception of government environmental information disclosure (EID) can promote rural residents’ pro-environmental behavior (PEB) using a questionnaire survey. Using Zhejiang province of China as a case study, we designed a four-stage mixed sampling method, which yielded 783 valid responses. We used ordinary least squares (OLS), an ordinal logit model and a mediation effect model to draw our conclusions. The results indicated that the EID had a positive impact on the PEB of rural residents. It is also evident that personal environmental concerns (PECs) play a partially mediating role between EID and PEB. Moreover, the impact of EID on PEB is heterogeneous in terms of residents’ age and workplace. This research contributes to insights into the promotion of guiding rural residents’ PEB and improving ecological environment management.

## 1. Introduction

Environmental pollution in rural areas has received increasing attention in recent years [1]. Industrial enterprises are incentivized to emit more pollutants to avoid pollution cleanup expenses and maximize revenues because environmental pollution is likely to go unrecognized or receive minimal fines in rural areas. Rural populations are forced to bear an environmental burden that is disproportionate to their level of economic growth or income [2]. However, rural residents have insufficient awareness of pro-environmental behavior (PEB) and improperly dispose of garbage, which greatly increases the difficulty of garbage collection and disposal [3]. Thus, rural residents’ PEB plays a critical role in environmental governance. Some studies have focused on macroeconomic factors affecting PEB, such as economic expansion [4] and environmental ethics education [5]. Most researchers show that micro perception and PEB have a positive relationship [6,7]. Some scholars believe that individuals with greater environmental knowledge are more enthusiastic about PEB [8,9].

At the same time, the government regards environmental information disclosure (EID) as an essential environmental governance tool. Environmental regulation tools can be divided into command and control regulation tools, market incentive regulation tools, and public participation regulation tools. The government regards EID as having the potential to reduce pollutants and carbon emissions and to improve environmental quality for a low-cost policy implementation [10,11]. The EID of the government is not only an important tool for the government to communicate environmental performance to the outside world but is also an effective way to improve the environment [10,11,12], which favours the role of enterprises’ EID in environmental governance [12]. In 2007, *Measures for the Disclosure of Environmental Information (for Trial Implementation)* were successively issued. In addition, *Government Information Disclosure Regulations* were officially implemented in 2019. The implementation of these laws and regulations clarifies the necessity of government EID [12]. EID has a positive impact on air pollution control [10]. Improving the level of pollution source information disclosure enhances cities’ ability to reduce sulfur dioxide emissions, confirming “target accountability” and “backward coercive effects” [11]. The media can raise public awareness by exposing a company’s carbon disclosure information through positive or negative reports [12]. Thus, negative media reports on companies’ carbon disclosure information can put pressure on the heavily polluting industries and play an important role in raising public awareness and environmental governance. Also, residents’ perceptions of government actions can positively influence their PEB [13]. However, little research has been done on rural residents’ perception of EID. We wonder if EID has a positive impact on PEB in rural areas, which can promote rural environment protection and help the government better use policy tools to achieve environmental governance. For this reason, this study is needed to explain the relationship between EID and PEB in order to make policy recommendations for the improvement of environmental protection and sustainable development.

Therefore, we investigated the impact of EID on PEB from the perspective of rural residents. We conducted a survey of rural residents in Zhejiang province using a four-stage mixed sampling method. This study uses the ordinary least squares (OLS) estimation and the ordered logit model for benchmark regression. To verify the robustness of the benchmark results, we adjusted the dependent variable, independent variable, regression model, and sample size. Considering that residents with stronger environmental preferences may be more inclined to fill out the questionnaire, we adopted the Heckman sample selection models, according to Quaglione et al. [14]. We also explore whether results vary by ages and workplaces. Based on the findings, we further investigated the mediating role of personal environmental concern (PEC).

There are two contributions in this paper. This paper examines the impact of rural residents’ perception of EID on PEB in developing countries from a micro level and clarifies the direct impact mechanism of EID on PEB. There are few studies from this perspective. At the same time, we discovered the mediating mechanism of PEC. This paper reveals that receiving information on environmental pollution increases concern for the environment which boosts rural residents’ PEB. We can emphasize intermediary mechanisms, especially raising residents’ environmental concerns, to improve the environment. This study sheds new light on how regional variability affects environmental governance.

This paper is organized as follows. Section 2 presents the EID of the government and PEB, reviews relevant research, and proposes the research hypotheses. Section 3 describes the survey and sampling design, data source, the selection of variables, and the ordered logit model. Section 4 evaluates the effect of the perception of EID on the PEB of rural residents and the estimated results of the mediating development of PEC on PEB. Finally, the paper concludes in Section 5 with policy insights.

## 2. Literature Review and Research Hypothesis

### 2.1. Influencing Factors of PEB at the Macro Level

Economic development promotes public environmental awareness and PEB at the macro level. The general public becomes wealthier as the economy improves, increasing the need for and ability to improve environmental quality [4]. The development of rural tourism has a certain relationship with the PEB of rural residents [15,16]. The implementation of a rural tourism destination strategy means managers have to improve local environmental management in order to increase tourist loyalty. Environmental pollution and public environmental preservation behavior have a positive relationship. Smog pollution has been proven to promote the consumption of energy-saving appliances by Chinese urban residents [17]. People are less likely to buy eco-friendly products and engage in sustainable behaviors when exposed to air polluteon. Air pollution induces negative emotions and inhibits people’s willingness to engage in PEB [18]. Policy tools have an impact on PEB [19,20,21]. The passage of legislation also has a favorable impact on environmental goals [19]. In addition, government-provided monetary and nonmonetary incentives to households can considerably reduce water consumption [20]. Social normative intervention as a policy tool has received much attention recently [21]. Structural background and cultural factors can affect people’s participation in PEB [22,23,24,25]. For example, social norms better predict PEB in Israeli people [22]. French speakers were found to place a higher value on the environment, probably because they have a stronger sense of collectivism and generosity [8]. In addition, the public’s access to environmental protection information has changed as technology has advanced. The growth and use of the internet has had a significant impact on knowledge exchange [26]. According to Ho et al. [27], substantial media output has a significant effect on PEB by increasing individuals’ attention to environmental news and support for social environmental activities. However, there is less research on EID and more research on its influencing factors in the extant literature.

### 2.2. Influencing Factors of PEB at the Micro Level

The influencing factors at the micro level are mostly concentrated on environmental knowledge [9,28,29,30], environmental perception [7,9,31,32], environmental protection attitudes [31,33,34], theory of planned behavior (TPB) [35,36,37] and personal habits [37].

Individuals with greater environmental knowledge are more enthusiastic about PEB. Furthermore, research has shown that the association between environmental knowledge and PEB is substantially more robust in private environmental behaviors than in their public counterparts [28]. Residents’ perceptions of sustainability-related climate directly impact on their participation in PEB [9]. In particular, the positive thoughts of homemakers about perceived behavioral control have a positive effect on guiding their recycling behaviors [32]. Concern for the environment and perceived consumer effectiveness promote a connection with nature. In both cases, consumer perceived effectiveness is a critical construct that directly impacts on green-choice behavior [32,38]. Residents’ attitudes toward the environment are reflected in their awareness of the importance of environmental protection and personal subjective perception. The perception and importance of environmental protection are the critical factors affecting personal environmental resource protection [29]. Moreover, the link between pro-environmental attitudes and pro-environmental activities is more vital when the opportunity cost is smaller [34].

Community participation is the most powerful predictor of PEB [28]. The public’s satisfaction will affect their PEB. The public’s contentment with the governments’ environmental protection efforts influences citizens’ water-saving behaviors [29]. Furthermore, life satisfaction has a significantly stronger and more substantial impact on high-cost environmental behaviors than it does on low-cost environmental behaviors [39]. In a solid waste disposal experiment, the vast majority of respondents felt there was a link between their well-being and appropriate waste disposal behavior [40]. Moreover, the pace of life, which differs by gender, affects PEB. Women with fast-paced lives tend to be more pro-environment [41]. Habits still seriously hinder Chinese urbanites from engaging in green behavior in all PEB [37]. An increase in family income promotes citizens’ individual PEB but reduces the possibility of public PEB [42]. Educational level is another essential factor in predicting PEB because it can raise citizens’ awareness of the beneficial external impacts of PEB [43].

Many studies examine the elements that influence residents’ PEB, most of them focus on the micro level, on human subjective perceptions and on subjective will, while few analyze the perceived influencing factors related to government policy tools.

### 2.3. EID and PEB

Information is becoming increasingly important in an era of rapid development. Research revealed that receiving information on battery electric vehicles increased the intention of purchasing [44]. Increasing health information can improve individual health outcomes [45]. In the fight against COVID-19, government actions to deliver messages have been associated with meaningful behavioral changes, such as wearing masks and washing hands [46]. Limited or missing environmental quality information has a particular impact on households’ housing choices, thereby affecting their exposure to biological pollution and household welfare. Domestic households are more exposed to pollution and are more harmed when there is a shortage of quality environmental information [47].

The EID of the government positively impacts environmental governance. As it encourages businesses to take proactive steps to reduce pollution, it also influences the impact of environmental governance through public interaction [48]. The association between EID and the PEB of the people was discovered to be conditional after thorough study. An open government drives civic engagement more effectively by addressing many aspects of data presentation, such as benchmark selection [49]. Residents’ PEB is affected differently by the availability of active and passive environmental information. Passive receipt of environmental information distributed by central and local governments, for example, encouraged public participation and support for a hypothetical urban river restoration project. In contrast, the role of active environmental information is frequently limited [50]. Based on the above analysis, this paper puts forward the following research hypothesis:

**Hypothesis** **1.** *Rural residents’ perception of the rural government EID has a significant positive impact on PEB*.

### 2.4. PEC and PEB

Many studies have confirmed that environmental concern does affect various behaviors of environmental protection, such as green-purchasing behavior [51], fuel consumption behavior [52], energy use [53], etc. Generally, environmental concern is essential to behaving in an environmentally friendly manner [54]. According to Rhead et al. [55], environmental concern is linked to environmental behavior, i.e., the more people care about the environment, the more likely they are to engage in environmentally responsible behavior, and vice versa [56]. According to cognitive consistency theory, attention to the environment will compel people to engage in appropriate environmental behavior. The more a person is aware of environmental issues, the more likely he or she is to encourage sustainable consumption habits [57]. Environmental problems are viewed as mediating variables in the majority of research studies. PEC has an intermediary influence on personal experience of environmental protection intention [58]. In addition, environmental concerns have been shown to mediate the relationship between waste sorting and green consumption behavior [22,38,47,59]. On the other hand, public environmental concerns have been proven to have no significant impact on people’s environmental participation [60]. There are still questions about the impact of PEC on PEB. The relationship between PEC and PEB, whether directly or through other variables, has also been confirmed by significant research, so we introduce the following research hypothesis:

**Hypothesis** **2.** *Rural residents’ PEC have an intermediary effect on their PEB.*

EID has a considerable positive impact on residents’ PEB, according to the literature [48,49]. However, if the motivation theory of avoiding responsibility is ignored, the release of government environmental data may induce the psychology of evading responsibility in some people, triggering the “free rider” effect and inhibiting residents’ PEB. In terms of research methodology, some studies employ the questionnaire survey approach, while others use the intermediary effect model to combine the micro and macro elements that influence residents’ PEB. This research thoroughly understands the value of EID and residents’ PEB based on the aforementioned investigation. As a result, the text examines the direct influence of EID on residents’ PEB as well as the indirect impact of residents’ environmental worries on their PEB using questionnaire data.

## 3. Materials and Methods

### 3.1. Questionnaire

#### 3.1.1. Survey Design

The first stage in determining the influence of EID on rural inhabitants’ PEB involved creating a questionnaire. We designed a four-part questionnaire and distributed it in the form of a paper questionnaire. The first section of the survey examines the respondents’ views on the local environment. The second section examines the respondents’ perceptions of government work and their understanding of it. The third section examines the respondents’ perceptions of environmental protection knowledge and PEC about the national strategy of village revitalization. Wang et al. and Foroughi et al. [61,62] used a five-point Likert scale to measure environmental knowledge, environmental awareness, and environmental concern. When Keren et al. studied environmental concern and social norms for recycling, they used a five-point and a six-point Likert-type scale items respectively [22]. We used a seven-point Likert scale in the third part of questionnaire. The fourth section examines the respondents’ age, gender, occupation, educational level, and family background. Kautish et al. looked at demographic characteristics of participants including age, gender, income, education, occupation, and marital status when examining the impact of consumer-perceived validity on their spending behavior [38].

#### 3.1.2. Sampling Design

China’s Zhejiang province has some experience in environmental governance, and there is certain reference significance to use Zhejiang province’s 11 prefecture-level cities as the research object. Thus, we investigated rural inhabitants’ attitudes and views on environmental protection in Zhejiang province, China, using a four-stage mixed sampling method to achieve randomness. Table A1 in Appendix A shows the sampling method. With the intention of analyzing representative data and to ensure diversity of the entire country, Kautish et al. selected one state capital from each of the eastern (E), western (W), northern (N), and southern (S) regions of the country to collect samples [38]. Li et al.’s research group divided China’s agricultural areas into five regions: north central region, northwest region, northeast region, east and southwest region according to the level of social and economic development and physical geographical characteristics [63]. In each region, one province was randomly selected. The sample counties, towns and administrative villages were randomly selected according to the order of economic development level. Geng et al. used a stratified random sampling method to select Xuzhou (northern city ranked sixth in private car ownership in Jiangsu province), Suzhou city (southern city ranked second), and Nanjing (middle city ranked first) as typical cities in the field research [6].

Thus, in the first phase, we used a stratified sampling method in order to guarantee that prefecture-level cities at every level of development were likely to be selected according to per capita GDP. In Geng et al.’s research [6], they randomly chose the communities or housing estates in the second stage, and then randomly chose the buildings and households in the third stage. The advantage is that as a simple random sampling method, it can ensure that all respondents have an equal chance of being selected to participate in this survey. According to his method, in the second stage, the method of simple random sampling was used to select one municipal district and county from each of the five cities. We utilized basic random selection in the third stage and used convenience sampling in the fourth stage. Foroughi et al. used non-probabilistic convenience sampling to investigate the determinants of hotel guests’ environmental behavior [62].

**Figure 1:**
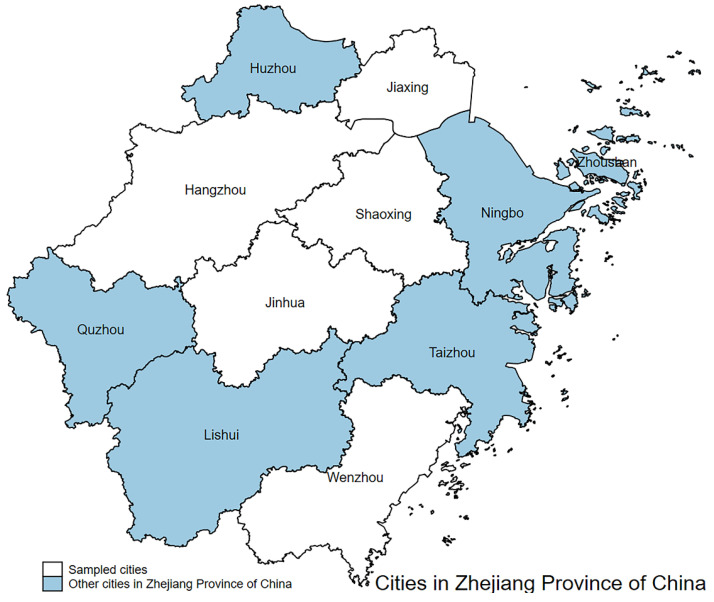
5 cities were selected from 11 prefecture-level cities using a stratified sampling method.

In order to ensure the accuracy of the research, the sample size must be determined scientifically. Assuming a confidence level of 95% (Z = 1.96) and a maximum allowable absolute error of 3.59%, we determined the final sample size to be 783. In the specific sampling, the prefecture-level cities were stratified according to per capita GDP, and the extraction indicators were required to cover 11 prefecture-level cities in Zhejiang province in the first stage as in Figure 1. We used stratified sampling to select 5 cities from a total of 11 prefecture-level cities in Zhejiang Province, China. Figure 1 shows that the five cities were Hangzhou, Jiaxing, Jinhua, Shaoxing, and Wenzhou. The result of the second stage simple random sampling was Qiantang District, Tongxiang City, Keqiao District, Wucheng District, and Lucheng District. The result of the third stage simple random sampling was Xiasha Street, Hezhuang Street; Fengming Street, Heshan Town; Huashe Street, Lanting Town; Chengdong Street, Jiangnan Street, Bailongqiao Town; and Wuma Street, Shuixin Street.

### 3.2. Variables and Data

Through the above process, we collected 807 questionnaires. In the 807 questionnaires, there were data beyond the valid range of options, lack of logical consistency and some missing data. After processing, 783 effective samples were finally obtained to analyze the impact of environmental information disclosure on rural residents’ PEB. The validity and reliability of the questionnaire passed the test. The definition and assignment of variables are shown in Table 1. The environmental behavior of rural residents in Zhejiang province is the dependent variable in this study. A collection of questions about rural people’ readiness to participate in PEB were asked in the questionnaire, and the number of environmental protection volunteers initiatives that rural residents engaged in revealed the enthusiasm of rural inhabitants for environmental protection. This paper will measure environmental information disclosure from the perspective of subjective perception, and the perception of government environmental information is the primary independent variable in this study. The focus of this paper is on the influence of EID on rural residents’ PEB. Rural people’s comprehension of the rural revitalization strategy, constructing a beautiful China, Zhejiang poetry and painting the Zhejiang gardens reflects their understanding of rural environmental policies and the publication of Zhejiang provincial government environmental data. The intermediary variable of this paper is the residents’ PEC, which transforms knowledge of rural revitalization, industrial prosperity, ecological livability, rural civilization, effective governance, and affluence into a comprehensive index. The control variables in this paper mainly included demographic variables and resource conditions. Demographic variables include age (*Age*), gender and marital status (*Sex*), registered residence (*Rr*), and future living style (*Ls*). Resource conditions include occupation (*Occ*), workplace (*Wp*), education level (*Edu*), and household average annual income (*Income*).

Table A3 in Appendix B reports the name, frequency, proportion, mean value, and standard deviation of the variables. Table A4 in Appendix B reports the correlation coefficients of the variables. The explanatory variables of this paper are the environmental behavior of residents in Zhejiang province and rural revitalization strategy information disclosure (*Rev*), beautiful China information disclosure (*Bea*), poetry and painting Zhejiang garden construction information disclosure (*Poe*), age, gender and marital status (*Sex*), occupation (*Occ*), workplace (*Wp*), the correlation coefficient of education level (*Edu*), family income (*Income*), registered residence (*Rr*) and future residence (*Ls*). Among them, rural revitalization strategy (*Rev*), beautiful China (*Bea*), poetry and painting Zhejiang garden construction information disclosure (*Poe*), age, gender and marital status (*Sex*), workplace (*Wp*), and annual family income (*Income*) are positively correlated with PEB; while occupation (*Occ*), education level (*Edu*), registered residence (*Rr*), and the way of living in the next three years (*Ls*) were negatively correlated with PEB.

### 3.3. Methodology

#### 3.3.1. OLS Estimation

When studying the impact of citizens’ attitudes toward the government on climate change, Ge et al. used the OLS method to find that respondents’ perceptions of climate change had a significant impact on their government’s attitudes towards climate change mitigation policies [64]. Martin et al. found a favorable connection between PEB and satisfaction using the OLS method [65]. The following method was used to determine the influence of environmental information sharing on residents’ PEB:

(1) $${behavior}_{i}=C_{1}+\beta_{1}{disclosure}_{i}+\beta_{2}X+\varepsilon$$

where i represents the individual rural residents, $behavior_{i}$ is the PEB of the *i*th rural resident, $disclosure_{i}$ is the openness of government environmental information reflected by the *i*th rural resident, and ε is a random disturbance term. Initially, the analysis of differences in PEB focused on sociodemographic factors, such as gender, age, education, marital status, place of residence, and personal economic situation [66]. For example, Christin et al. demonstrated gender differences in personal life rhythms for PEB [41]. The increase in family income will promote citizens’ individual PEB [42]. Educational level is an essential factor in predicting PEB because it can raise citizens’ awareness of the beneficial external impacts of PEB [60,67]. In addition, people who are active outdoors are more likely to feel the effects of environmental pollution [68]. Therefore, age (*Age*), gender and marital status (*Sex*), occupation (*Occ*), workplace (*Wp*), education level (*Edu*), family income (*Income*), registered residence (*Rr*), and future residence (*Ls*) are controlled in this research.

#### 3.3.2. Ordered Logit Model

When researching environmental information disclosure, PEB, and other challenges, some researchers have used the logit model [6,69]. The advantage of this method is that stated variables can be used for many classifications. The choice of explanatory variables and explained variables in this research is based on questionnaire data, and the explained variable selection gradient does not match the value requirements of the general binary logit regression model. As a result, to investigate the impact of EID on residents’ PEB, this work designed an ordered logit model.

Liu et al. employed the logit method to show that healthy behaviors are positively associated with cycling to commute [69]. Geng et al. used multiple logit methods to prove that green environmental motivation is a necessary condition to ensure the stability of green travel behavior [6]. In this paper, the PEB of residents is selected as the explained variable, and the explained variable is transformed into a natural logarithm $\ln\frac{p}{1-p}$, recorded as Logit(P): where *P* is the probability of respondents’ participation in environmental protection, and the model can be expressed as:

(2) $$Logit(p)=\ln\frac{p}{1-p}=C_{2}+\beta_{3}disclosure_{i}+\beta_{4}X+\mu$$

where i represents the individual rural residents, $disclosure_{i}$ is the core explanatory variable, and $X_{4}$ is the control variable, which is composed of age (*Age*), gender and marital status (*Sex*), occupation (*Occ*), workplace (*Wp*), education level (*Edu*), family income (*Income*), registered residence (*Rr*), and future residence (*Ls*). $C_{2}$ is a constant term, $\beta_{3}$ is the coefficient of the core explanatory variable, $\beta_{4}$ is the coefficient of the control variable, and μ is a random disturbance term.

#### 3.3.3. Mediation Effect Model

**Figure 2:**
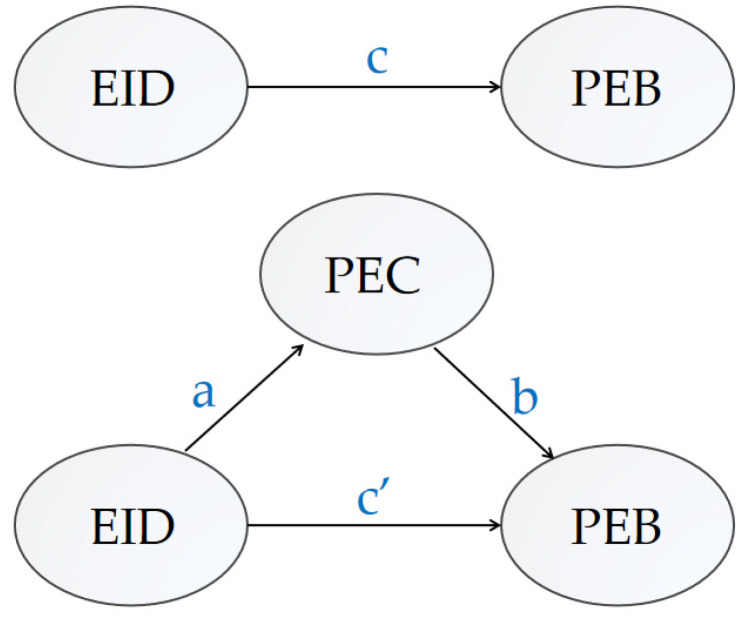
Schematic diagram of the mediation effect of PEC.

Mediating effect analysis is an important step to test whether a variable becomes a mediator and to what extent it plays a mediating role. Many studies have explored the mediating role of environmental concerns [8,47,58]. For example, Saari et al. proved that environmental knowledge and risk perception influence individuals’ sustainable consumption behavior through environmental concerns [8]. In this paper, the PEC of residents is selected as the mediating variable, and the model can be expressed as:

(3) $$behavior_{i}=cX+e_{1}$$

(4) $$concern_{i}=aX+e_{2}$$

(5) $$behavior_{i}=c\prime X+bconcern_{i}+e_{3}$$

where i represents the individual rural residents, $behavior_{i}$ is the dependent variable PEB, and X is the independent variable EID and the control variables. $concern_{i}$ is the mediating variable. Figure 2 shows the mechanism by which EID affects PEB. The coefficient a represents the effect of EID acting on the PEC, and the coefficient b represents the effect of PEC acting on PEB. The two constitute the indirect effect of the relationship between the variables in the figure. The coefficient *c*’ represents the effect of EID on PEB after controlling for PEC, that is, the direct effect between EID and PEB. The total effect between variables is equal to the direct effect plus the indirect effect, thus, $c=ab+c^{\prime }$. In this paper, the sobel method was used to test the mediating effect.

## 4. Results

### 4.1. Benchmark Regression

Table 2 shows the results of the benchmark regression. Relevant variables are not transformed. The OLS regression result under common standard error is reported in the first column of Table 2. The OLS regression result with robust standard error is shown in the second column. For ordered logit regression, the third column employs the same explanatory variables and control variables. The fourth column shows the ordered logit model’s regression outcome under robust standard error.

The findings suggest that the government’s revelation of information significantly positively impacts the rural residents’ PEB. The greater the degree of EID by the government, the more it boosts the PEB of rural residents. The government’s timely exposure of environmental policies may increase residents’ awareness of pollution. This result is consistent with the result of Chen et al. [56]. The revelation of pollution index data may raise residents’ understanding of the need to participate in environmental protection, thus stimulating PEB. The influence coefficients of the four columns in the regression findings are varied. In the ordered logit regression, the influence coefficient of EID is strong. The standard deviation of the openness of the primary explanatory variable is smaller under the robust standard error than under the ordinary standard error.

The OLS regression results demonstrate that age has a substantial positive impact on the PEB of rural people among the control variables, implying that the older the residents are, the more active they are in PEB. The selection of control variables in this paper is similar to that of Yang et al. [70]. Yin et al. demonstrated that public environmental concerns have no significant impact on their environmental engagement [60], considering gender, age, and income levels as control variables. Also, Chen et al. studied the relationship between environmental information and public participation, gender, age, education level and income included in the control variables, and they were hardly noticeable [50]. In this article, we added control variables to ensure the consistency of the estimates, but it is difficult for us to guarantee the validity of the estimates.

### 4.2. Robustness Check

The robustness test is conducted by gradually increasing the control variables, with the EID serving as the independent variable and the PEB of rural residents serving as the dependent variable. Table 3 displays the results. The first column shows the regression results when age, gender and marital status, occupation, workplace, and education are controlled. The annual household income is presented as the control variable in the second column based on the first. Table 3 introduces registered residence (*Rr*) and living style (*Ls*) control variables in the third and fourth columns. The analysis results show that when controlling for age (*Age*), gender and marital status (*Sex*), occupation (*Occ*), workplace (*Wp*), education level (*Edu*), family income (*Income*), registered residence (*Rr*), and living style (*Ls*), the explanatory variables are also available. There is still a significant positive correlation between PEB and EID. The PEB of rural residents in Zhejiang province is positively influenced by the information disclosure of the “rural revitalization strategy”, indicating that the results are robust. The relationship between PEB and the age of rural residents is still significantly positively correlated with the increase in the control variables, meaning that the age of rural residents has a positive impact on PEB.

The robustness test was conducted using the variable substitution approach, with the EID serving as the explanatory variable and the PEB of rural residents as the explanatory variable. Table 4 displays the results. The first and second columns employ the method of substituting the fundamental independent variables, with the creation of “beautiful China” (*Bea*) and the development of a “poetry and painting Zhejiang” garden (*Poe*) replacing the openness of the Zhejiang rural government to the “rural revitalization strategy”. The PEB of rural inhabitants is replaced by the willingness to engage in environmental protection in the third column of Table 4, replacing dependent variables. After removing the independent variables, the regression results demonstrate that the influence of EID is still substantial at 1%, and the results are stable. The rural government impact influences the “beautiful China” construction’s information disclosure, followed by the “rural revitalization strategy”. After substituting the dependent variable, the impact of EID on rural inhabitants’ willingness to conserve the environment is significant at the 1% level, indicating that the results are consistent.

The fourth column of Table 4 adopts the method of changing the sample size. Considering the differences in the degree of information disclosure of the municipal government, we selected the samples from Jiaxing city, Zhejiang province, for regression, with 159 samples. After reducing the sample size, the impact of EID on rural residents’ PEB is significant at the 5% level. The impact coefficient is lower than the benchmark regression, indicating that the positive impact of EID on rural residents’ PEB has a weak effect in Jiaxing. This may be due to the low degree of EID in Jiaxing, and that rural residents in Jiaxing pay less attention to the environment.

The ordered probit model is used in the first column of Table 5 to examine the robustness of the influence of EID on rural residents’ PEB. Since Puhani [71] indicates that the Heckman model gives more robust estimation results, it controls for collinearity. Previous studies have found that it is easy to make mistakes in sample selection, leading to endogenous problems [72,73]. Rural residents who are not sensitive to environmental information may be excluded when research focuses only on residents’ perceptions of EID This leads to sample selection bias and affects the consistency of the estimator. To solve this problem, we adopted the Heckman two-step estimation method [63]. The second column of Table 5 is the two-step estimation method’s regression result for the sample selection model. The regression outcome of the sample selection model calculated using the MLE estimation approach is in the third column. The fourth section contains the regression results of the sample selection model computed using the MLE estimation method with robust standard error. The regression findings of the control interaction term (willingness * disclosure) are in the fifth column.

After replacing the model with an ordered probit model for testing, the impact of EID on rural households’ PEB is still considerable at 1%. Under the OLS and ordered logit models, the impact coefficient is lower than the regression result. The sample selection model’s results demonstrate significance at the 1% level, showing that the outcome is steady. The more available government environmental information is, the more it can promote the PEB of rural communities. Rural inhabitants’ age has a substantial impact on their PEB, indicating that the PEB of rural residents will be stimulated as they grow older. The controlled interaction item (willingness * disclosure) has a negative, but not significant impact on residents’ PEB.

### 4.3. Heterogeneity

Combining the above analysis results, it is clear that the age of residents has a considerable favorable effect on their PEB. As a result, heterogeneity analysis was carried out on the age of the rural residents, and the results are displayed in Table 3. The regression findings of the questionnaire data of inhabitants aged 16–20, 20–30, 30–40, 40–50, 50–60, and over 60 are represented in Table 6 from the first column to the sixth column. Except for individuals over 60 years old, the data demonstrate that government disclosure of environmental policy has a significant positive impact on rural residents’ PEB. This could be due to adults over the age of 60 having a lower level of education and higher level of illiteracy. For such elderly residents, the government should adopt corresponding approaches to stimulate PEB.

Environmental pollution may be seen differently by residents in different workplaces. Residents who work outside may have a more negative opinion of pollution and pay closer attention to environmental legislation. People who work outside, for example, can experience the local air quality more than residents working indoors within a given period. Therefore, this paper conducted a group regression on the workplace to observe the relationship between the PEB of the respondents in indoor and outdoor workplaces and the disclosure of government environmental information. The seventh column of Table 6 reports the impact of the EID on residents working indoors on their PEB. The eighth column reports the impact of the EID of residents working outdoors on their PEB. The analysis shows that the PEB of residents working indoors or outdoors is positively correlated with the revelation of environmental information, which is significant at 1%. This may be because the data used in this paper have certain limitations. In the samples of indoor work, most occupations are school students, who have a high level of awareness of environmental protection, thus, improving the participation rate of PEB.

### 4.4. Intermediary Effect

This article uses the rural residents’ environmental concern (PEC) as an intermediary variable to examine whether EID can affect PEC, thereby stimulating rural residents’ PEB. Numerically, the first column in Table 7 shows that EID has a positive promoting effect on rural residents’ PEC, with a coefficient of 5.375, which is significant at 1%. The improvement of EID can promote the growth of rural residents’ PEC, which is consistent with the previous theoretical expectation. The second column shows that rural residents’ PEC has a positive effect on their PEB, with a coefficient of 0.243 and EID also has a positive effect on rural residents’ PEB, with a coefficient of 0.010. This means that EID promotes residents’ PEB by improving rural residents’ PEC, indicating support for Hypothesis 2.

The Sobel method was used to test the mediating effect. Table 8 shows that the results passed the significance test at the 5% level.

## 5. Conclusions and Implications

### 5.1. Conclusions

This research aimed to determine the effect of EID on PEB and the mediating effect of PEC. It has been demonstrated that EID has a significant positive impact on the PEB of rural residents, and there is a mediating effect of PEC on PEB. Our research highlights the direct effects of EID on PEB and environmental governance, and the importance of PEC’s indirect impact on PEB and environmental governance. The results showed relatively consistent promoting effects.

The findings demonstrate that EID has an impact on rural inhabitants’ PEB. The empirical analysis yielded four results. First, the release of government environmental data had a significant positive impact on rural residents’ PEB. The government’s disclosure of vital environmental policies, in particular, allows rural residents to participate in environmental conservation while also stimulating their PEB. Second, rural residents’ PEC plays an intermediary role in their PEB. The government successfully increases rural residents’ PEC through EID and stimulates their PEB by exposing them to environmental information and environmental protection measures that they generally disregard. People must first recognize the importance of environmental protection in their subjective consciousness to create good habits related to environmental protection in daily activities and actively participate in environmental protection initiatives. Third, except persons over the age of 60, the government’s publicizing of vital environmental regulations has a considerable positive impact on residents’ PEB. As a result, strategies that encourage the PEB of older rural residents effectively support the improvement of the rural ecological environment. Fourth, there is a substantial positive link between PEB and EID, whether working indoors or outside.

This paper enriches the study of EID on PEB from the individual perspective. The conclusions of this paper further support Chen and Cho [50], while this paper further discusses the impact mechanism. Previous studies have found that EID can reduce pollution emissions [11] and reduce pollution losses [10], while few studies are from the micro perspective and mechanism. The existing research on EID is mainly from the objective point of view, while research from the subjective cognitive perspective provides a new perspective. We extended the study of PEC [55,56] and found that it can also affect PEB as an intermediary variable. This helps to understand the significance of PEC in theory and draws more attention to the importance of strengthening the impact of PEC in policy making.

There are still some shortcomings in the following areas. Because of the characteristics of the questionnaire and data of this paper, the generalization of the conclusions of this paper may be limited. Although we attempted to reduce the impact of endogeneity, we have not found a more appropriate identification strategy, such as the appropriate instrumental variable. The regulatory issues affecting residents’ PEB are not discussed in depth. During heterogeneity analysis, there are obvious apparent differences in sample size between indoor and outdoor areas due to data limitations, which reflects the investigation’s limitations and may impact on the analysis. On this basis, if a follow-up investigation or expansion of the sample size can be carried out, the experimental results can be made more representative.

### 5.2. Implications

The government and inhabitants must work together to support the establishment of ecological civilization and sustainable development to reach the pinnacle of green growth. Only by fully utilizing the role of EID as the third wave of environmental regulation can we compensate for the environmental pollution caused by rural residents’ lack of environmental awareness and poor infrastructure construction, thus improving the rural environment and promoting the construction of ecological civilization. As a result, to fully use EID’s potential and encourage rural communities to participate in environmental preservation, this article makes the following three recommendations.

First, the government should ensure the implementation of EID laws and regulations and urge the government to disclose environmental information promptly, which will help stimulate rural residents’ PEB directly and indirectly, to achieve rural environmental governance. Second, the government should pay attention to the intermediary mechanism. The government’s attention to the PEC of rural residents will also positively affect the residents’ PEB. Third, the importance of age heterogeneity in increasing popularity should be considered. Environmental pollution control, environmental protection expertise, and PEB are sensitive topics for rural inhabitants of various ages and occupations. As a result, the government should formulate distinct publicity strategies for groups based on their heterogeneity, which are more effective and favorable to increasing popularity.

## Figures and Tables

**Table 1:** Definitions and operations of variables.

Variable Name	Variable Definition	Variable Assignment
Behavior	Environmental protection behavior of rural residents	
Disclosure	Government environmental information disclosure	
Rev	Publicity of Rural Revitalization Strategy	1 = Very little 7 = Very well
Bea	Open strategy of beautiful China	1 = Very little 7 = Very well
Poe	Poetry and painting Zhejiang Grand Garden Construction open	1 = Very little 7 = Very well
Concern	Environmental concerns of rural residents	1 = Very little 7 = Very well
Age	Age	1 = 16–20 years old 2 = 21–30 years old 3 = 31–40 years old 4 = 41–50 years old 5 = 51–60 years old 6 = Over 60 years old
Sex	Gender and marital status	1 = Female married 2 = Female unmarried 3 = Male married 4 = Male unmarried
Occ	Occupation	1 = Farming 2 = Enterprises with pollution discharge 3 = Non polluting enterprises 4 = Student 5 = Social organization or group 6 = Party and government organs and institutions not engaged in environmental protection work 7 = Party and government organs and institutions working in environmental protection system 8 = Other
Wp	Workplace	1 = Indoor 2 = Outdoor
Edu	Education level	1 = Primary school and below 2 = Junior high school 3 = High school/technical secondary school/Technical School 4 = Junior college 5 = Bachelor degree 6 = Master’s degree or above
Income	Average annual household income	1 = Less than 50,000 2 = 50,000–100,000 3 = 100,000–200,000 4 = 200,000–300,000 5 = 300,000–400,000 6 = 400,000–500,000 7 = More than 500,000
Rr	Registered residence	1 = Local 2 = Foreign migration within 3 years 3 = Foreign migration more than 3 years 4 = Out of town
Ls	Living style in the next three years	1 = Long term settlement 2 = Family visit or vacation 3 = Hope to settle down for a long time 4 = Move out 5 = Will not come again

Notes: PEC is a comprehensive indicator, and other indicators are not weighted.

**Table 2:** Benchmark regression results.

	OLS		Ordered Logit	
	(1)	(2)	(3)	(4)
	Common Standard Error	Robust Standard Error	Common Standard Error	Robust Standard Error
Disclosure	0.290 *** (0.0505)	0.290 *** (0.0418)	0.313 *** (0.0432)	0.313 *** (0.0421)
Age	0.232 *** (0.0863)	0.232 ** (0.100)	0.0529 (0.0696)	0.0529 (0.0701)
Sex	0.116 (0.0850)	0.116 (0.0886)	0.0560 (0.0694)	0.0560 (0.0706)
Occ	−0.0515 (0.0408)	−0.0515 (0.0515)	−0.00955 (0.0337)	−0.00955 (0.0353)
Wp	0.0137 (0.298)	0.0137 (0.375)	0.0569 (0.242)	0.0569 (0.250)
Edu	0.00480 (0.0880)	0.00480 (0.0716)	−0.0202 (0.0711)	−0.0202 (0.0709)
Income	0.0111 (0.0620)	0.0111 (0.0666)	0.0129 (0.0511)	0.0129 (0.0532)
Rr	−0.0783 (0.0958)	−0.0783 (0.0767)	−0.0718 (0.0785)	−0.0718 (0.0784)
Ls	0.0764 (0.0991)	0.0764 (0.0764)	0.109 (0.0794)	0.109 (0.0781)
Constant	−0.560 (0.709)	−0.560 (0.729)		
Observations	752	752	752	752
R-squared	0.064	0.064		

Notes: The *t* value is reported in parentheses below; **, and *** represent the 5%, and 1% significance levels, respectively.

**Table 3:** Robustness test for stepwise addition of control variables.

	(1)	(2)	(3)	(4)
	OLS Behavior	OLS Behavior	OLS Behavior	OLS Behavior
Disclosure	0.293 ***	0.292 ***	0.289 ***	0.290 ***
	(0.0419)	(0.0420)	(0.0417)	(0.0418)
Age	0.225 **	0.232 **	0.230 **	0.232 **
	(0.100)	(0.101)	(0.100)	(0.100)
Sex	0.112	0.114	0.117	0.116
	(0.0858)	(0.0887)	(0.0885)	(0.0886)
Occ	−0.0495	−0.0545	−0.0550	−0.0515
	(0.0492)	(0.0503)	(0.0503)	(0.0515)
Wp	0.0148	0.00540	0.00628	0.0137
	(0.370)	(0.375)	(0.375)	(0.375)
Edu	0.0136	0.00998	0.0145	0.00480
	(0.0712)	(0.0712)	(0.0710)	(0.0716)
Income		0.0117	0.0128	0.0111
		(0.0665)	(0.0666)	(0.0666)
Rr			−0.0572	−0.0783
			(0.0749)	(0.0767)
Ls				0.0764
				(0.0764)
Constant	−0.544	−0.552	−0.482	−0.560
	(0.707)	(0.727)	(0.723)	(0.729)
Observations	754	753	753	752
R-squared	0.062	0.063	0.064	0.064

Notes: The *t* value is reported in parentheses below; **, and *** represent the 5%, and 1% significance levels, respectively.

**Table 4:** Robustness test for replacement of independent variables, dependent variables, and sample size.

	(1)	(2)	(3)	(4)
	OLS Behavior	OLS Behavior	OLS willingness	OLS Jiaxing’s
Disclosure			0.226 ***	0.152 **
			(0.0372)	(0.0730)
Bea	0.314 ***			
	(0.0452)			
Poe		0.270 ***		
		(0.0443)		
Age	0.230 **	0.234 **	0.0891	−0.0990
	(0.0984)	(0.102)	(0.0601)	(0.231)
Sex	0.111	0.114	−0.157 ***	0.0467
	(0.0885)	(0.0889)	(0.0601)	(0.148)
Occ	−0.0560	−0.0640	0.00774	−0.0762
	(0.0512)	(0.0521)	(0.0286)	(0.0563)
Wp	0.0258	−0.0636	0.253	−0.635
	(0.372)	(0.375)	(0.212)	(0.451)
Edu	0.0151	0.0391	0.00104	−0.0175
	(0.0690)	(0.0699)	(0.0615)	(0.188)
Income	−0.0029	0.0200	0.0844 *	0.0272
	(0.0654)	(0.0662)	(0.0452)	(0.111)
Rr	−0.0743	−0.0758	0.0157	−0.239 **
	(0.0776)	(0.0776)	(0.0678)	(0.1100)
Ls	0.0769	0.0735	−0.0166	−0.182
	(0.0758)	(0.0755)	(0.0734)	(0.1680)
Constant	−0.627	−0.305	3.486 ***	2.032
	(0.721)	(0.721)	(0.500)	(1.287)
Observations	752	752	751	159
R-squared	0.074	0.064	0.083	0.065

Notes: The *t* value is reported in parentheses below; *, **, and *** represent the 10%, 5%, and 1% significance levels, respectively.

**Table 5:** Robustness tests for changing the benchmark regression into the probit model and the Heckman model.

	(1)	(2)	(3)	(4)	(5)
	Ordered Probit	Heckman Behavior	Heckman Behavior	Heckman Willingness	Heckman Behavior
Disclosure	0.188 *** (0.0244)	0.292 *** (0.0503)	0.292 *** (0.0503)	0.292 *** (0.0243)	0.299 *** (0.0821)
Age	0.0503 (0.0410)	0.233 *** (0.0858)	0.233 *** (0.0858)	0.233 * (0.140)	0.234 *** (0.0862)
Sex	0.0361 (0.0413)	0.119 (0.0846)	0.119 (0.0846)	0.119 * (0.0691)	0.119 (0.0848)
Occ	−0.00966 (0.0206)	−0.0495 (0.0406)	−0.0495 (0.0406)	−0.0495 (0.0380)	−0.0495 (0.0406)
Wp	0.0520 (0.148)	0.0393 (0.297)	0.0393 (0.297)	0.0393 (0.406)	0.0391 (0.297)
Edu	−0.00919 (0.0402)	0.00674 (0.0875)	0.00674 (0.0875)	0.00674 (0.0689)	0.00668 (0.0875)
Income	0.00846 (0.0308)	0.0105 (0.0617)	0.0105 (0.0617)	0.0105 (0.0613)	0.0108 (0.0618)
Rr	−0.0444 (0.0451)	−0.0784 (0.0952)	−0.0784 (0.0952)	−0.0784 (0.0759)	−0.0784 (0.0952)
Ls	0.0609 (0.0446)	0.0771 (0.0985)	0.0771 (0.0985)	0.0771 ** (0.0346)	0.0769 (0.0986)
W × D					−0.0011 (0.0113)
Constant		−0.624 (0.708)	−0.624 (0.708)	−0.624 (0.819)	−0.629 (0.710)
Observations	752	750	750	750	750
R-squared					

Notes: The *t* value is reported in parentheses below; *, **, and *** represent the 10%, 5%, and 1% significance levels, respectively.

**Table 6:** Heterogeneity test results.

	(1)	(2)	(3)	(4)	(5)	(6)	(7)	(8)
	Aged 16–20	Aged 20–30	Aged 30–40	Aged 40–50	Aged 50–60	Over 60	Indoors	Outdoors
Disclosure	0.245 *** (0.0629)	0.321 *** (0.0808)	0.217 * (0.116)	0.258 *** (0.0986)	0.490 * (0.281)	0.924 (1.211)	0.260 *** (0.0458)	0.440 *** (0.103)
Age							0.285 *** (0.109)	−0.156 (0.212)
Sex	0.162 (0.121)	0.167 (0.147)	−0.136 (0.188)	−0.039 (0.189)	0.803 (0.651)	−0.381 (1.592)	0.129 (0.0994)	0.0492 (0.228)
Occ	−0.155 (0.115)	0.300 ** (0.121)	−0.113 (0.102)	−0.038 (0.0580)	−0.522 (0.322)	0.344 (0.407)	−0.055 (0.0561)	0.053 (0.137)
Wp	1.664 (1.912)	−0.462 (0.560)	1.686 * (1.005)	−0.514 (0.345)	−1.943 (1.274)	4.160 (3.690)	−0.077 (0.406)	−0.394 (0.843)
Edu	−0.096 (0.143)	−0.003 (0.152)	0.148 (0.214)	0.281 ** (0.133)	0.733 (0.739)	−0.556 (2.106)	0.014 (0.0794)	−0.092 (0.149)
Income	0.123 (0.0755)	0.062 (0.111)	−0.384 (0.255)	−0.515 *** (0.127)	0.568 (1.045)	0.285 (1.325)	−0.019 (0.0733)	0.171 (0.162)
Hrl	−0.113 (0.107)	−0.049 (0.193)	−0.001 (0.230)	−0.164 (0.152)	0.641 * (0.362)	−0.434 (1.846)	−0.087 (0.0805)	−0.030 (0.250)
Fr	0.114 (0.116)	0.188 (0.136)	0.271 (0.304)	−0.315 (0.231)	−0.132 (1.306)	−0.303 (1.501)	0.062 (0.0857)	0.230 (0.193)
Constant	−1.302 (2.296)	−1.988 (1.653)	−0.423 (1.249)	2.673 *** (0.928)	−0.283 (3.221)	−6.192 (5.407)	−0.386 (0.802)	−0.623 (1.862)
Observations	217	218	91	151	56	19	642	110
R-squared	0.110	0.137	0.155	0.143	0.161	0.280	0.062	0.169

Notes: The *t* value is reported in parentheses below; *, **, and *** represent the 10%, 5%, and 1% significance levels, respectively.

**Table 7:** Mediating effects affect test results.

	(1)	(2)
	Concern	Behavior
Disclosure	5.375 ***	0.010 ***
	(0.4958)	(0.0038)
Concern		0.243 ***
		(0.0547)
Age	4.409 ***	0.191 **
	(0.8461)	(0.0882)
Sex	1.391 *	0.111
	(0.8366)	(0.0858)
Occ	0.017	−0.061
	(0.4003)	(0.0410)
Wp	−3.528	0.104
	(2.9446)	(0.302)
Edu	0.479	0.003
	(0.8611)	(0.0882)
Income	0.383	0.010
	(0.6112)	(0.0626)
Hrl	−1.815 *	−0.044
	(0.9435)	(0.0968)
Fr	−1.897*	0.074
	(0.9740)	(0.0999)
Constant	66.723 ***	−1.294 *
	6.9951	(0.759)
Observations	742	742
R-squared	0.2117	0.0750

Notes: The *t* value is reported in parentheses below; *, **, and *** represent the 10%, 5%, and 1% significance levels, respectively.

**Table 8:** Sobel test results.

	Coefficient	Std. Err.	Z	*p* > |Z|
Sobel	0.0529	0.0209	2.5300	0.0114
Goodman-1	0.0529	0.0209	2.5200	0.0117
Goodman-2	0.0529	0.0208	2.5410	0.0111

## Data Availability

Not applicable.

## Appendix A

Table A1 and Table A2 show the four-stage mixed sampling method, sampling unit, sampling indicators and sampling results and GDP per capita of 11 cities in Zhejiang province at the first stage of sampling.

**Table A1:** Population sampling method.

Stage	Sampling Unit		Sampling Indicators	Sampling Method	Sampling Results
The first stage	Prefecture-level administrative region		Urban development	Stratified sampling	Hangzhou, Jiaxing, Shaoxing, Jinhua, Wenzhou
The second stage	County-level administrative region	Zoning code	Simple random sampling	Qiantang District, Tongxiang City, Keqiao District, Wucheng District, Lucheng District	
The third stage	Township/town/street	Partition encoding	Simple random sampling	Xiasha Street, Hezhuang Street; Fengming Street, Heshan Town; Huashe Street, Lanting Town; Chengdong Street, Jiangnan Street, Bailongqiao Town; Wuma Street, Shuixin Street	
The fourth stage	Respondents	Number of residents	Chance sampling	/	

**Table A2:** Basis for the first stage of sampling: per capita GDP of 11 cities in Zhejiang province in 2019.

Cities	GDP per Capita (Ten Thousand Yuan)
Lishui	66,936
Quzhou	71,087
Wenzhou	71,225
Jinhua	81,224
Taizhou	83,555
Huzhou	102,593
Jiaxing	112,751
Shaoxing	114,561
Zhoushan	116,781
Ningbo	143,157
Hangzhou	152,465

## Appendix B

Table A3 and Table A4 presents the descriptive statistic and the correlation coefficients for the whole sample.

**Table A3:** Descriptive statistics including variable description, frequency, proportion, mean, standard deviation.

Variable	Description of Variables	Frequency	Proportion	Mean Value	Standard Deviation
Behavior	Minimum: 0			1.41	2.35
	Maximum: 26				
Rev	Minimum: 1			4.42	1.72
	Maximum: 7				
Bea	Minimum: 1			4.35	1.77
	Maximum: 7				
Poe	Minimum: 1			3.74	1.83
	Maximum: 7				
Age	16–20 years old	230	29.4%	2.54	1.40
	21–30 years old	228	29.1%		
	31–40 years old	94	12.0%		
	41–50 years old	156	19.9%		
	51–60 years old	56	7.2%		
	Over 60 years old	19	2.4%		
Sex	Female married	186	24.1%	2.38	1.07
	Female unmarried	266	29.2%		
	Male married	159	20.6%		
	Male unmarried	162	21.0%		
Occ	Farming	91	11.7%	4.60	2.18
	Enterprises with pollution discharge	12	1.5%		
	Non polluting enterprises	62	7.9%		
	Student	371	47.5%		
	Social organization or group	15	1.9%		
	Party and government organs and institutions not engaged in environmental protection work	50	6.4%		
	Party and government organs and institutions working in environmental protection system	6	0.8%		
	Other	174	22.3%		
Wp	Indoor	669	86.4%	1.14	0.34
	Outdoor	105	13.6%		
Edu	Primary school and below	50	6.4%	3.87	1.36
	Junior high school	119	15.2%		
	High school/technical secondary school/Technical School	107	13.7%		
	Junior college	130	16.6%		
	Bachelor degree	356	45.6%		
	Master degree or above	19	2.4%		
Income	Less than 50,000	80	10.2%	3.20	1.47
	50,000–100,000	180	23.0%		
	100,000–200,000	244	28.7%		
	200,000–300,000	161	20.6%		
	300,000–400,000	50	6.4%		
	400,000–500,000	28	3.6%		
	More than 500,000	38	4.9%		
Rr	Local	651	83.1%	1.40	0.94
	Foreign migration (within 3 years)	21	2.7%		
	Foreign migration (more than 3 years)	41	5.2%		
	Out of town	70	9.0%		
Ls	Long term settlement	555	71.0%	1.56	0.95
	Family visit or vacation	57	7.3%		
	Hope to settle down for a long time	136	17.4%		
	Move out	29	3.7%		
	Won’t come again	5	0.6%		

**Table A4:** Correlation coefficient table.

Variables	(1)	(2)	(3)	(4)	(5)	(6)	(7)	(8)	(9)	(10)	(11)	(12)	(13)
(1) behavior	1.000												
(2) rev	0.210	1.000											
(3) bea	0.233	0.810	1.000										
(4) poe	0.220	0.637	0.716	1.000									
(5) concern	−0.043	0.187	0.139	0.091	1.000								
(6) age	0.118	0.021	0.018	0.113	−0.010	1.000							
(7) sex	0.007	−0.018	−0.012	−0.040	−0.069	−0.290	1.000						
(8) occ	−0.025	0.056	0.064	0.082	0.093	0.074	−0.071	1.000					
(9) wp	0.039	−0.087	−0.079	0.018	−0.066	0.422	0.023	−0.218	1.000				
(10) edu	−0.045	0.131	0.106	0.001	0.065	−0.652	0.212	−0.009	−0.433	1.000			
(11) income	0.008	0.136	0.147	0.066	0.000	−0.217	0.162	0.101	−0.203	0.307	1.000		
(12) rr	−0.044	−0.053	−0.052	−0.077	0.014	−0.158	0.108	−0.027	−0.069	0.151	0.058	1.000	
(13) ls	−0.005	−0.018	−0.017	−0.052	0.000	−0.268	0.117	−0.116	−0.145	0.293	0.121	0.316	1.000
